# A model of spontaneous mouse mammary tumor for human estrogen receptor- and progesterone receptor-negative breast cancer

**DOI:** 10.3892/ijo.2014.2657

**Published:** 2014-09-17

**Authors:** LIXIANG ZHENG, BUGAO ZHOU, XIANMING MENG, WEIFENG ZHU, AIREN ZUO, XIAOMIN WANG, RUNDE JIANG, SHIPING YU

**Affiliations:** 1 School of Basic Medicine, Jiangxi University of Traditional Chinese Medicine, Nanchang, Jiangxi 330004, P.R. China; 2 Jiangxi University of TCM Science and Technology, Nanchang, Jiangxi 330004, P.R. China; 3 Key Laboratory of Modern Preparation of TCM Ministry of Education, Jiangxi University of Traditional Chinese Medicine, Nanchang, Jiangxi 330004, P.R. China; 4 Department of Scientific Research, Jiangxi University of Traditional Chinese Medicine, Nanchang, Jiangxi 330004, P.R. China

**Keywords:** spontaneous mice mammary tumor model, pathology typing, marker

## Abstract

Breast cancer (BC) is the most frequently malignancy in women. Therefore, establishment of an animal model for the development of preventative measures and effective treatment for tumors is required. A novel heterogeneous spontaneous mammary tumor animal model of Kunming mice was generated. The purpose of this study was to characterize the spontaneous mammary tumor model. Histopathologically, invasive nodular masses of pleomorphic tubular neoplastic epithelial cells invaded fibro-vascular stroma, adjacent dermis and muscle tissue. Metastatic spread through blood vessel into liver and lungs was observed by hematoxylin eosin staining. No estrogen receptor (ER) or progesterone receptor (PR) immunoreactivity was detected in their associated malignant tumors, human epidermal growth factor receptor-2 (HER-2) protein weak expression was found by immunohistochemistry. High expression of vascular endothelial growth factor (VEGF), moderate or high expression of c-Myc and cyclin D1 were observed in tumor sections at different stages (2, 4, 6 and 8 weeks after cancer being found) when compared with that of the normal mammary glands. The result showed that the model is of an invasive ductal carcinoma. Remarkably in the mouse model, ER and PR-negative and HER2 weak positivity are observed. The high or moderate expressions of breast cancer markers (VEGF, c-Myc and cyclin D1) in mammary cancer tissue change at different stages. To our knowledge, this is the first report of a spontaneous mammary model displaying colony-strain, outbred mice. This model will be an attractive tool to understand the biology of anti-hormonal breast cancer in women.

## Introduction

Breast cancer (BC) is the most common malignancy in women and the mortality rate has been continuously increasing over the past 30 years. About one million women worldwide are diagnosed with BC every year (1,2). So it is very important to prevent tumorigenesis and treat cancer. Establishment of an animal model for the development of preventative measures and effective mammary cancer treatment is needed. Mouse models are excellent tools to understand the natural biology of breast cancer. Since human breast cancers are clustered into several phenotypes (subtypes) based on grade and molecular markers, a good animal model for a subtype is one which mimics most subtype characteristics morphology, molecular markers, metastatic pattern, grade, hormone-dependency, parity/pregnancy status and so on (3–5).

Kunming mice are closed colony mice and the largest number in production capacity in China. They are widely used in biology, pharmacology, toxicology and other areas of scientific research in China (6–11). Chinese scientist detected Kunming mice in one of the heterozygous breeding females (12–16). In our study, we found that Kunming female species after breeding can develop spontaneous breast cancer in 10–12 months (average 11.5 months). The pathological diagnosis was primarily invasive ductal carcinoma (17,18).

But BC is a biologically heterogeneous disease and patients with the same diagnostic and clinical prognostic profiles can have markedly different clinical outcomes. This difference is possibly caused by the limitation of our current taxonomy of breast cancers, which groups molecularly distinct diseases into clinical classes based mainly on morphology. Molecular profiling has provided biological evidence for heterogeneity of breast cancer through the identification of intrinsic subtypes. Analysis of gene expression data suggest that breast cancers can be divided into molecular subtypes which have distinct clinical features, with markedly differing prognosis and clinical outcomes. Therefore, immunohistochemical (IHC) markers have been used as surrogates in subtyping breast cancer. The updated IHC subtype definition was given as luminal A [ER^+^ and/or progesterone receptor (PR^+^), HER2^−^], luminal B (ER^+^ and/or PR^+^, HER2^+^), HER2^+^/ER^−^ (ER^−^, PR^−^, HER2^+^), basal-like (ER^−^, PR^−^, HER2^−^, CK5/6^+^), and unclassified (negative for all five markers) (19,20).

In the present study, in order to understand the histopathological and molecular characteristics of the model we elucidated the pathogenesis of breast cancer to identify specific therapeutic targets. We have subclassified the breast cancers in the spontaneous breast cancer model of Kunming mice (21,22). We used IHC staining to determine the expression of ER-α, PR, HER-2/neu and to identify intrinsic subtypes using formalin-fixed, paraffin-embedded tumor blocks, western blot analysis of c-Myc, cyclin D1 and VEGF gene expression. We also determined how relevant the model is for human breast cancer associations between tumor subtypes and tumor characteristics.

## Materials and methods

### Mouse model

Female Kunming species mice (female), 6–8 generation, average 12 month-old) were purchased from Shanghai Experimental Animal Center. The animal studies were in compliance with the university rules of conduct and adhered to the principles of Institutional Animal Care and Use Committee Guidebook (http://en.wikipedia.org). The use, management and welfare of the study animals met the Chinese Animal Care Regulations for Captive and Laboratory Animals as outlined in the 1988 National Regulation on Laboratory Animal Research, issued by the Ministry of Science and Technology. The animals were housed in accordance with Guidelines for the Care and Use of Laboratory Animals in scientific research (Chinese National Science Academy) in registered animal facility. The animals were maintained in Cabin type isolators at standard environmental condition (temperature 22–25°C, humidity 40–70%) with 12:12 dark/ light period. The mice were palpated on the breast every 3 days, trained technicians palpated all mammary glands of all animals and noted the location and size of all nodules, using standard technique (13–15). The mice were split to breed after cancer being found. Tumor weight was estimated by palpation. No precise quantitative guidelines such as the acceptable upper limit of tumor burden was available, since the adverse effects on the host depend on the biology of the tumor, the site and mode of growth.

In total 398, mice aged 11 to 12 months, were used in this study. Of the 89 cancer-bearing mice, spontaneous breast cancer was found with an average of 307 days after birth (306 to 448 days). After euthanasia, mammary glands and spontaneous breast cancer tissues were collected from each cancer-bearing animal at difference stages. The control mammary glands were collected from 18 month-old mice in two abdominal mammary glands. The final volume of cancer tissue was measured by the method of water immersion (23).

### Histopathological analysis

Histopathological evaluations were done. After euthanasia, mammary tumors and all organs were collected in 10% buffered formalin (liver, lungs, kidneys, heart, spleen, brain, pancreas, bone, adrenals, small and large intestine, uterus, ovary, cervix and urinary bladder). Formalin-fixed and paraffin-embedded tissues were cut at 5 μm thickness, stained with haematoxylin and eosin following standard procedure and examined under a light microscope.

### Immunohistochemistry

Sections from formalin-fixed, paraffin embedded tumors were cut and mounted on slides. After deparaffinization in xylene, slides were rehydrated through graded series of alcohol and placed in Tris buffer. Endogenous peroxidase activity was blocked with 3% hydrogen peroxidase and methanol. Commercially available antibodies to ER, PR, and Her2/neu, were used in the study. After tissue pretreatment including steam antigen retrieval and protein block, slides were incubated with antibody followed by incubation with horseradish peroxidase conjugated HRP. 3,3′-Diaminobenzidine tetrahydrochloride (DAB) chromogen was used for visualization of the antibody/ enzyme complex. Appropriate positive and negative controls were included with each IHC run. All cases were studied for ER, PR and Her2 antibodies. Staining results were assessed by two of the authors on a double headed microscope. A case was considered positive for a given marker only when both observers agreed upon its specificity and distribution. Nuclear or membrane immunostaining for ER, PR and Her2 was evaluated counting a total of 1,000 cells in 10 representative fields at high magnification (×200). The number of immunopositive cells was expressed as a percentage (mean, median, minimum and maximum values).

The intensity of ER, PR and Her2 immunoreactivity was graded on a scale of 0 to 3, in which 0, no reactivity; 1, weak; 2, moderate; and 3, strong reactivity.

### Western immunoblot analysis

Protein was extracted from fresh-frozen biopsy specimens from Kunming mice mammary carcinomas and normal mammary glands. Each sample was placed in 2-ml Eppendorf safe-lock tubes and immersed in Laemmli buffer for lysis. After incubation on ice for 20 min, tissue lysates were clarified for 10 min at 12,000 × g at 4°C, denatured at 95°C for 5 min, and stored at −80°C until needed. Protein concentrations were normalized using BCA reagent according to the manufacturer’s protocol (Pierce, Rockford, IL). For electrophoresis, protein extracts from fresh-frozen mammary cancer were subjected to SDS-PAGE in 8% polyacrylamide gels according to Laemmli *et al* (24). Electrophoresis was stopped when the tracker dye reached the end of gels.

For western immunoblots, electrophoresed proteins were transferred to nitrocellulose membranes and blocked in phosphate-buffered saline, 0.05% Tween-20 (PBS-T), plus 5% skim milk for 1 h or overnight. The membrane was then incubated with the cyclin D1, VEGF, c-Myc HER-2/ neu polyclonal rabbit anti-human antibody at 1:1,000 dilution (DakoCytomation) in PBS-T plus 2% skim milk for 2 h, washed five times with PBS-T, and incubated for 1 h with peroxidase-conjugated goat anti-rabbit secondary antibodies (Sigma-Aldrich) in PBS-T plus 2% milk. Membranes were visualized with ECL^+^ after incubation with anti-mouse or rabbit secondary antibody (1:5,000) (GE Healthcare).

### Statistical analysis

Statistical analysis was performed by Statistical Package for Social Sciences (SPSS) Version 17.0. All of the statistical tests were two-tailed and P-value less than 0.05 was considered as statistically significant.

## Results

### Animals

**Figure 1:**
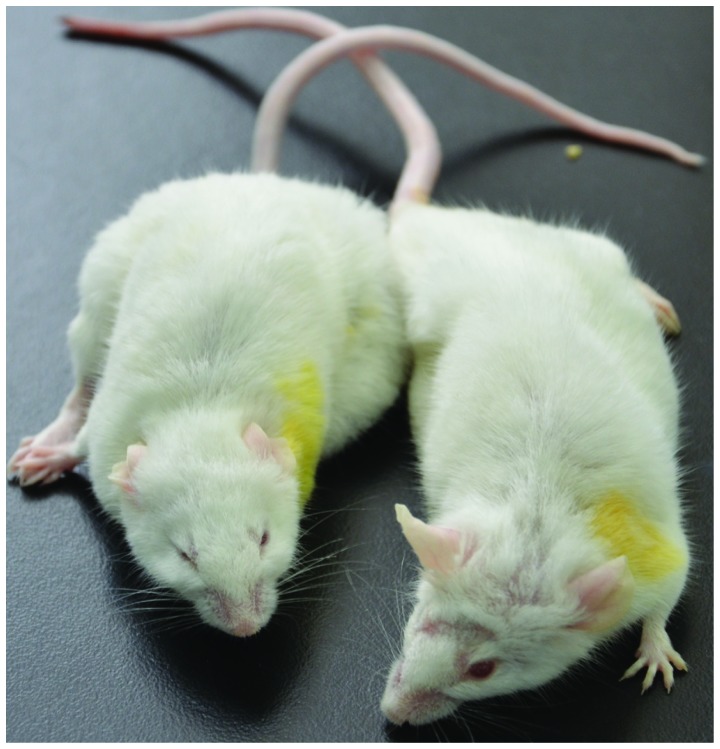
Kunming heterozygous female mice showing mammary tumors.

**Figure 2:**
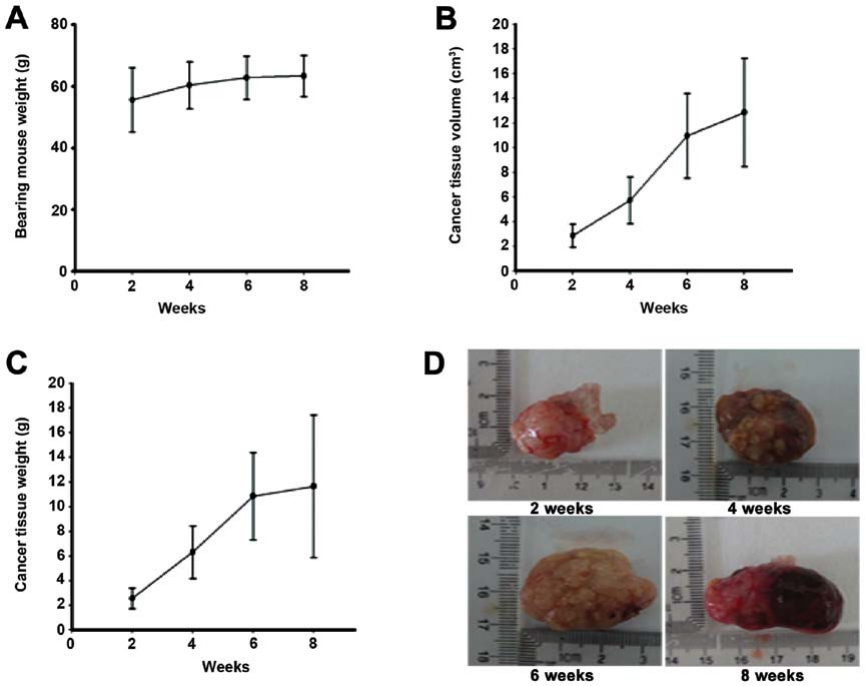
Biological features of spontaneous mammary tumor mice (tumor-bearing mice). (A) The weight of tumor-bearing mice. (B) Volume of different stages of cancer tissue. (C) Weight of different stages of cancer tissue. (D) Morphological characteristics of different stages of cancer tissue.

Kunming mammary tumors were observed in female breeding mice, mostly after delivering the 6–8th litter (about average 11.5 months), after stopping breeding they continued to rear 1–8 weeks. Among the 398 females bred, 89 mice developed mammary carcinoma (incidence rate 25%). The age of tumor occurrence was on average about 13.5 months (range 12–16 months), the life span of mammary tumor mice was about 8 weeks (6–10 weeks). The females that were not mated did not develop tumors during their life span (data not shown). The tumors were unilateral i.e., developed either in right or left side mammary glands and were observed only in the 2nd or 5th or both mammary glands (Fig. 1). The tumor grew rapidly in the initial stage and reached 10% body weight in about 2 weeks. At the time, the body weight of the bearing mice was increased. During 4–6 weeks, the mice got thin gradually, and the body weight did not increase obviously (Fig. 2A). At the stage that they were euthanasized for ethical reasons the average body weight was about 62 g, cancer weight was about 12 g and the tumor-size was about 13 cm^3^ (Fig. 2B and C). The life span was about 62 days.

### Foci develop in mammary cancer of Kunming mice

About 4 weeks after cancer development, some of neoplastic mass appeared as multi-lobes with ulceration on the skin surface. On palpation, fluid thrills were observed in each lobe. Exteriorized tumor mass revealed multi-lobes at 6, 8 weeks, each lobe had multiple solid nodules enclosing a central necrosed tissue with foul smelling inflammatory exudates and clotted blood (Fig. 2D).

Histologically, the expansile tumor mass pushed through the overlying dermis and was found infiltrating the muscle layer underlying the mammary gland and had indistinct borders at 6 weeks. The tumor mass was similar to tubular epithelial-like morphology with differentiated lobes (Fig. 2D).

### Histopathological analysis

**Figure 3:**
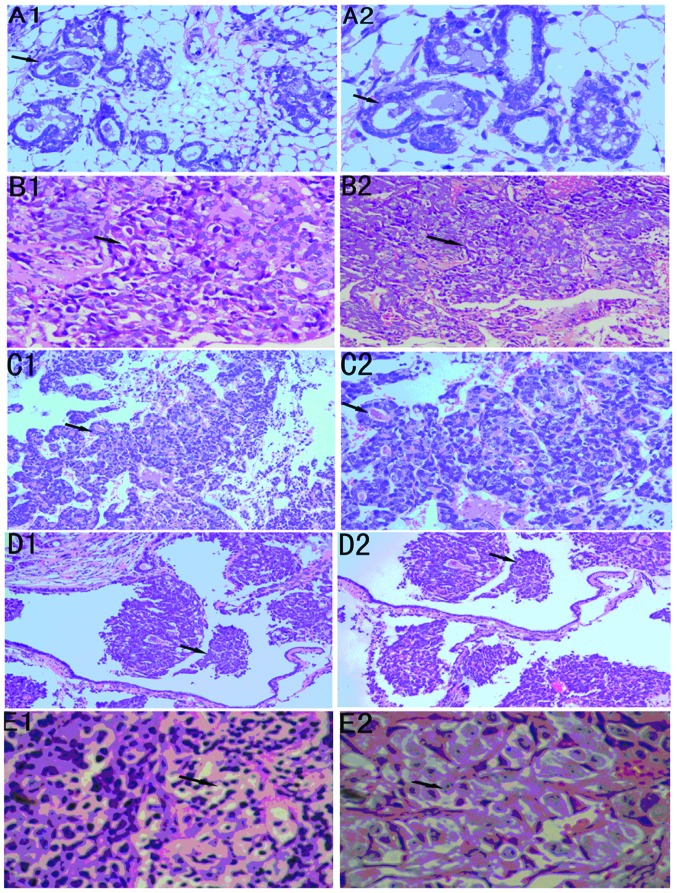
Mouse and human breast cancer pathology at different stages. (A) Histopathology of normal mouse mammary gland, the epithelial cells are vacuolated with cytoplasmic accumulation of fat droplets (A1, ×200; A2, ×400). (B) At two weeks cancer tissue cells are lined with hyperchromatic nuclei and rarely prominent nucleoli. Few myoepithelial cells are admixed in the hyperplastic epithelium (B1, ×100; B2, ×200). (C) At four weeks cancer tissue was high-grade comedo ductal carcinoma *in situ* and there were few mammary gland ducts. Highly pleomorphic cuboidal to oval cells with abundant eosinophilic granular cytoplasm, round nuclei and prominent nucleoli (C1, ×100; C2, ×200). (D) At eight weeks cancer tissue was in a distended duct with central necrosis. Lymphocytes and plasma cells infiltrate the periductal stroma (D1, ×200; D2, ×400). (E) Intermediate-grade ductal carcinoma *in situ* in woman with proliferation of pleomorphic cuboidal cells with moderate eosinophilic cytoplasm, oval to elongate nuclei, and single prominent nucleoli (E1, ×200; E2, ×400).

Resected specimens were sectioned at 5 μm continuously and microscopically examined after hematoxylin and eosin staining. The morphologically normal ducts are surrounded by a well-developed basement membrane with a terminal duct entering a cluster of lobules (Fig. 3A1 and A2). Such a lobular-like structure, however, is similar to the lobular development found in the mouse mammary glands of early to mid-pregnant mice and also appear similar in structure to the terminal duct lobular unit in a normal adult on white adipose cells (25–27). One marked difference between the mouse mammary gland and human breast tissue is that the mouse tumor and ducts are surrounded by a thin fibroblastic stroma adjacent to the adipocytes that form the majority of the gland and which are often very close to the epithelial structures (Fig. 3B). In contrast, in humans, the lobules have extensive extracellular connective tissue stroma, therefore the adipocytes are less proximal to the ductal epithelium human breast (28,29).

The cytological atypia is a distinct morphological change typically found in the center of the tumor at 2–4 weeks in cancer tissue, the tumor cells now appear pleomorphic, showing a moderate variation in size and shape (Fig. 3B1 and B2) and nuclear pleomorphism. The majority of cells are mononuclear with macrophage morphology. An increased vessel density is found in the vicinity of the tumor adjacent to the areas with dense leukocytic infiltration (Fig. 3C1 and B2). High density of leukocytic infiltration is often observed in the human tumors (28,29). The majority of the ducts in the mammary glands that carry these early carcinomas are still morphologically normal, except for focal areas in which there is mild ductal epithelial hyperplasia with a small increase in the number of cell layers (data not shown).

By 6 to 8 weeks the primary tumors progressed to the advanced carcinoma stage, termed late carcinoma. At this stage the tumor is composed of solid sheets of epithelial cells with little or no remaining acinar structures visible (Fig. 3D1 and D2). The malignant cells in the tumor have marked variation in cellular and nuclear size and shape with vesicular nuclei and prominent nucleoli.

The patterns in the human tissue are heterogeneous, some solid sheets of cells filled the terminal duct lobular unit surrounded by a well-formed basement membrane and connective tissue. At this late carcinoma stage, multiple tumor nodules as well as ductal hyperplasia were found throughout the mammary gland (data not shown). Kunming mouse tumors at this stage have many characteristics that are similar to those of a human breast cancer classified as poorly differentiated invasive ductal carcinoma. The tubular structure disappeared. Multiple area of necrosis with infiltration of phlegmonosis material appeared in between and within mammary glands duct at 8 weeks. These infiltrates are composed of cells with the morphology of macrophages, fibroblasts and neutrophils. There was evidence of necrosis at higher magnification (Fig. 3E1 and E2). An increased vascularity was also observed at these sites.

**Figure 4:**
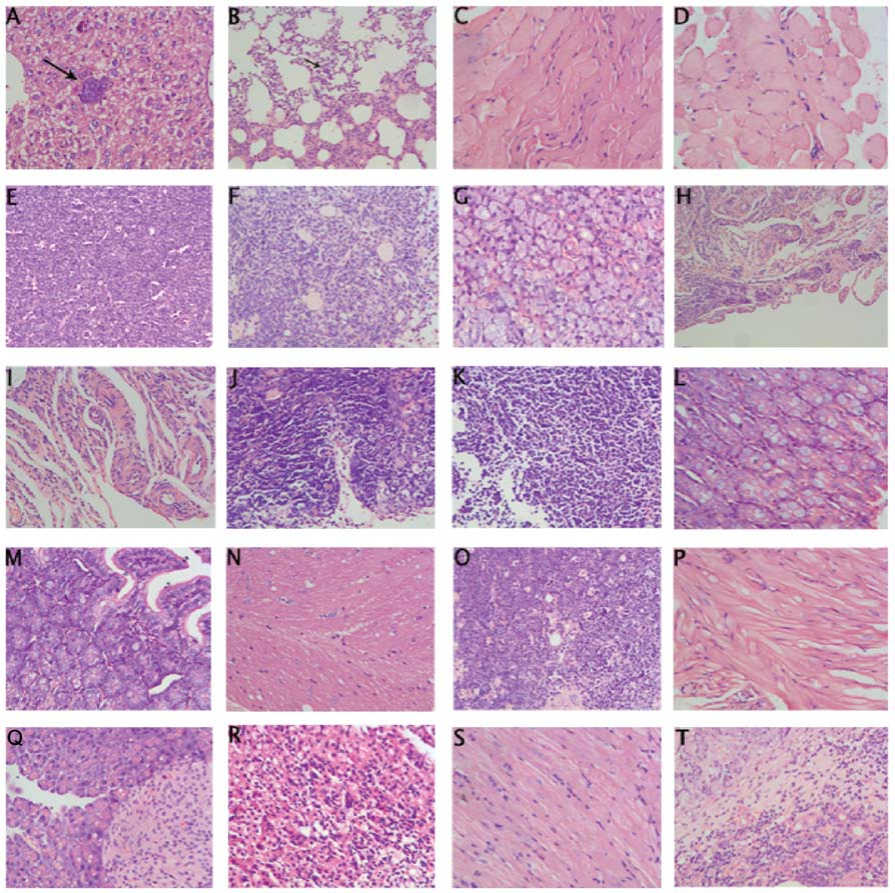
Tumor metastasis and non-tumor metastasis tissue. i) Metastatic foci in liver showing luminal epithelial-like morphology as in the primary (A, arrow, ×200). ii) Metastatic foci in lung by single or multiple layers of pleomorphic epithelial cells (B, arrow, ×200). iii) Non-tumor metastasis tissue (C, sternum; D, pectoralis major; E, lymphaden; F, ovary; G, submandibular gland; H, uterus; I, cervix; J, thymus; K, spleen; L, large intestine; M, small intestine; N, cerebrum; O, omentum majus; P, bladder; Q, pancreas; R, adrenal glands; S, heart; T, liver; magnification, ×200).

Neoplastic tubular epithelial cells metastasized into the liver and lungs (Fig. 4A and B, long arrows). No metastatic foci in bone, heart, adrenal, kidney, brain, intestines and pancreas were detected (Fig. 4C–T). Nodular-type tumors were found in the liver and lungs. Single foci of tumor emboli consisting of pleomorphic neoplastic cells spreading through lung blood vessels were present in the lungs. Large sized pleomorphic neoplastic cells were found in live follicles. Nodular-type tumors consisting of pleomorphic tubular epithelial cells were found attached to inner wall of the lung aorta (data not shown).

### Immunochemistry of ER/PR and HER2 expression

Breast cancer is a heterogeneous disease with a variety of pathological entities and varied clinical behavior and different molecular alterations driving its growth, survival and response to treatment. Estrogen-α and progesterone-receptor (ER and PR) expression is routinely used to determine the prognosis of human breast cancers, expression loss being associated with poor prognosis (20). To further examine the relevance of the Kunming mouse model for studies on human breast cancer, the expression of ER-α and PR in the tumors of Kunming mice at difference time was examined with IHC.

**Figure 5:**
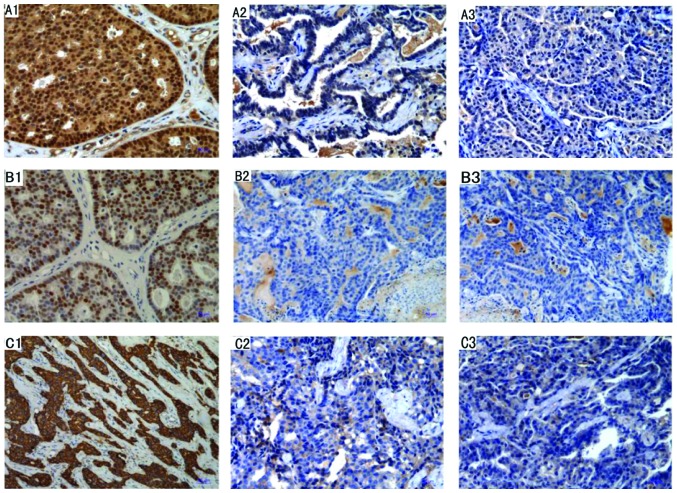
Immunohistochemical evaluation of ER-α, PR, HER-2/neu in mouse and human female DCIS. Strong and diffuse nuclear expression of (A1) ER-α, (B1) PR in human mammary cancer, but the model mouse cancer cells with no ER-α (A2 and A3), PR (B2 and B3) expression. Human mammary tumor with strong membraneous stain (3+) of human epidermal growth factor receptor 2 (HER2) (C1). Mouse cancer cell showing weak membrane (1+) HER-2/ neu expression (C2 and C3). Immunoperoxidase-DAB. Bar, 10 μm.

The IHC result indicated that both nuclear ER-α and PR immunoreactivity and nuclear colocalization were not present in the difference stages of breast cancer cells (Fig. 5A2, A3, B2 and B3). There was virtually no ER-α, PR-positive cells in the tumor, except in the residual ductal structures inside or adjacent to the tumor, but HER2 was weakly positive (Fig. 5C1 and C2).

All tumors were ER^−^, PR^−^, but HER2 was weakly positive [20% cells with nuclear staining (Fig. 5C1 and C2). Tumors with ER^−^ and/or PR^−^ are known to have higher risk of mortality after diagnosis compared to women with ER^+^ and/or PR^+^ disease. Several clinical studies have demonstrated women with HR^−^ tumors have poor survival advantage by treatment with adjuvant hormonal and/or chemotherapeutic regimens (30–33).

### Marker expression detected by immunoblot analysis

To investigate maker gene expression in normal mammary tissue and cancer tissue, 8 normal mouse mammary glands and 8 malignant mouse mammary tumors were subjected to western immunoblot analysis. The antibodies were also used to probe western blots of protein lysates of age-matched mammary glands and tumors at different stages of progression that had been carefully dissected away from the surrounding tissues.

A 62-kD band, corresponding to c-Myc (Abcom), was observed in tumor samples at different stages (Fig. 4A). Differences in band intensity were observed at comparable total protein loads. In particular, blotting of neoplastic tissues at four different stages produced four different types of bands, the strongest intensity was at 2 weeks (Fig. 4, lane 2), and intermediate intensity at 4 to 8 weeks (Fig. 4, lanes 3–5). Normal mammary tissues had a clearly detectable band of weak intensity (Fig. 4, lane 1).

The expression of cyclin D1 (Santa Cruz Biotechnology, Santa Cruz, CA) is found in the developing tumor and the mammary gland of Kunming mice. There is an increase in the tumor cell expression while at the same time the expression in normal ducts decreases. Anti-cyclin D1 antibodies recognizes a 43-kD protein in mammary tumors at various ages (Fig. 4C). The blotting at 2 to 4 weeks were the brightest at comparable total protein intensity (Fig. 4C, lanes 2 and 3), but weaker at 6 to 8 weeks. Normal mammary tissue was the weakest (Fig. 4C, lanes 4 and 5).

The expression of VEGF gene was not different (Fig. 4B). The band intensity in 6 to 8 weeks was the strongest in all the bands (Fig. 4B, lanes 4 and 5), but weaker at 2 to 4 weeks (Fig. 4B, lanes 2 and 3). The normal bands were the weakest (Fig. 4B, lane 1).

## Discussion

Breast cancer is the most frequent cancer in women (23% of all cancers), and it ranks second overall when both sexes are considered together (34). So it is very important to investigate breast carcinogenesis and cancer progression. Animal models are powerful tools to analyze the mechanism of the induction of human breast cancer. Most tumors are ductal infiltrating carcinomas expressing estrogen and progesterone receptors. The majority of the genetically modified mouse breast cancer models as well as most spontaneous, chemically or mouse mammary tumor virus (MMTV)-induced mammary tumors in mice do not express ER and PR, or if they do (some MMTV models), they are pregnancy-dependent (35). Although the application of transgenic technology in mice to study the progression of mammary cancer has proven extremely powerful to understand important principles of tumorigenesis and evaluating response to therapy, few of these models reflect the complexity of human breast cancers, especially their progression to metastasis as these models lack many aspects of human cancers. A lack of understanding about the natural history of the disease is a major contributory factor to this limitation (2–6). So a successful animal model that develops spontaneous mammary tumors that resemble human breast cancer in many aspects is needed.

Kunming mouse (KM mouse) is a genetically heterogeneous mouse. So the mice bear genetically heterogeneous spontaneous mammary tumors, similarly to randomly selected groups of cancer patients. Therefore, the model is an alternative to other rodents. To our knowledge, this is the first report to use closed-population Kunming mouse to make spontaneous breast cancer.

KM mice are a unique closed population of laboratory mice in China. They are widely used in pharmacology, toxicology and other experiments (6–11). Some scholars in China have made all-round studies and thought that KM mice is different from NIH and other international renowned out-bred, in that it is a out-bred mouse with unique genetic traits. The ancestor of Kunming mice was traceable from Western Europe *Mus Musculus domesticus* and was introduced to China’s Yunnan province. The ancestor of KM mice was polluted by *Mus Musculus domesticus* paternal genetics and gradually evolved into a different one from other out-bred populations with unique genetic characteristics. Experimental animals in a closed group require more than 5 years of closed breeding, random mating breeding method, production and reproduction (6–16). The species average age was 11.5 months at cessation of births. Those continuing to feed for 2–3 weeks suffered spontaneous breast cancer. On pathological diagnosis, the model is invasive ductal carcinoma (17,18).

Ductal carcinoma is the most common histological category of malignant breast tumors, lobular carcinoma is the second major type while medullary carcinoma is a relatively rare entity. On clinical diagnosis, the various presentations are classified on the basis of morphological and molecular examination. Prognosis is defined according to a number of parameters, tumor size and grade, the presence/absence of estrogen and/or progesterone receptors, HER2/neu (HER2, c-erbB2) protein, vascular or perineural tumor invasion (33,34).

Our HIC data show that all the different stages of cancer cells were ER^−^, PR^−^ and HER2 weak positive. So the sub-classification of the spontaneous breast cancer was luminal B. Most mice (80%) tend to have ER^−^, highly aggressive mammary tumors; thus, the spontaneous breast cancer model may be particularly suitable as an animal model of human hormone-resistance breast cancer.

HER-2/neu is a cell-membrane receptor tyrosine kinase, normally involved in the signal transduction pathways leading to cell growth and differentiation (2). Approximately 15–20% of breast cancers have amplification of the HER-2/neu gene or overexpression of its protein product (33). So the weak expression of Her2 resulted in tumor cell morphological differences among different stages of Kunming tumor-bearing mice in cancer tissue and partial necrosis in 6–8 weeks of tumor cells.

Most of the model displayed metastases to the liver and lungs. The tumors predominantly had luminal/tubular epithelial-like morphology (Fig. 4A and B). But other organs were free of metastasis. The spleens were enlarged at 6 to 8 weeks. The result showed hematogenous spread almost exclusively to the lungs, in contrast to human tumors, which show regional lymph node involvement with preferential spread to the liver.

In recent years, understanding of the underlying biological mechanisms of carcinogenesis and the altered molecular events has led to the identification of novel molecular targets and development of targeted therapies. Targeting the pathways that promote or sustain growth and invasion of carcinoma cells is critical to effective treatment of breast cancer. A better understanding of the biology of ER, PR-negative breast cancer is therefore needed. In this study, we investigated the common chromosomal amplifications found in human breast cancer, such as c-Myc and cyclin D1 (37–39).

**Figure 6:**
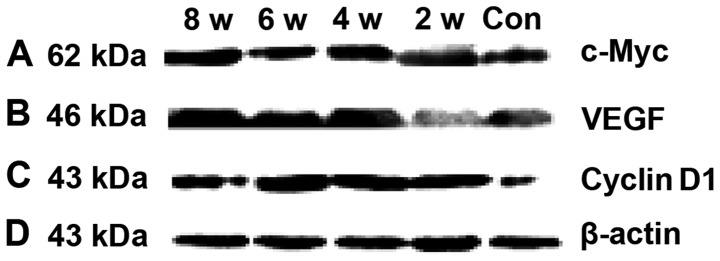
Intensity of bands indicates the expression level of the corresponding molecular markers by western immunoblot with the Santa Cytomation anti-Myc, -VEGF, -cyclin D1 antibody in 6 Kunming mouse mammary tumors. (A) c-Myc expression is weaker in normal mammary gland (Con), moderate to higher expression in 2 to 4 weeks than normal, but are the strongest in 6–8-week Kunming mouse mammary tumors. (B) VEGF band is weaker in Con, but at 2–8 weeks are gradually stronger in mammary tissues. (C) Cyclin D1 expression is weaker in Con while it is the strongest at 6 weeks in mammary tissues, moderate to higher expression at 2, 4, 8 weeks compared to normal.

The blotting result showed that c-Myc expression was significantly elevated in all the tumors tissues. Prior studies have examined Myc expression in breast cancers and the basal breast cancer subtype exhibits enrichment for a c-Myc transcriptional gene signature (36–39); so the hight expression of c-Myc in Kunming mice leads to proliferation of spontaneous breast cancer cells, and the cancer volume is greatly enlarged (Fig. 2D). Following tumor growth, the cancer needs additional nutrition, for new blood vessels formation. Thus, the expression of VEGF was increased significantly (Fig. 2D and 6B).

The differences in c-Myc expression allowed reciprocal studies on induction or repression of the metastatic phenotype by manipulation of c-Myc expression and its downstream targets. Clearly human breast cancers that overexpress c-Myc may still metastasize if other factors override its function (38,39). Our *in vivo* assays demonstrated that expression of c-Myc will support increased growth of those few metastatic cells that escape the inhibitory function of Myc by means of other mutations or by changes in gene expression (Fig. 6A). This phenomenon can help us explain why some Kunming mouse breast cancer cells metastasize to the lungs and the liver.

Several converging studies have suggested that c-Myc can be involved in the activation of cyclins (D1, D2, E1 and A2), cyclin-dependent kinases (CDK4), and in the downregulation of cell cycle inhibitors. The expression of Myc and CCND1 constitutes an early and transient event. The regulation of human CCND1 by progestins may be more complicated, while it has been suggested that PR regulates CCND1 expression by non-genomic mechanisms (36). The high expression of cyclin D1 may be related with this mechanism.

Loss of estrogen and progesterone receptor gene expression has been found in 30% of human breast cancers, and this condition is associated with less differentiated tumors and poor clinical outcome. Similarly, overexpression of ErbB2/neu and cyclin D1 has been found in ~20% of cases and this also correlates with poor prognosis (40,41). Remarkably, these phenomena seem to be recapitulated in the model with loss of ER and PR and weak expression of Her2/neu and overexpression of cyclin D1, suggesting a common pathway to malignancy between mammary cancers in mouse and human.

In the present study we performed a detailed histological and molecular marker analysis that showed many similarities to the histology of human tumors. We also analyzed a series of biomarkers associated with poor prognosis in human breast cancer. Remarkably in the Kunming mouse model, loss of estrogen and progesterone receptors and low expression of Her2/neu and overexpression of c-Myc, cyclin D1 and VEGF were observed, which is recapitulated in a manner similar to that observed in human breast cancer with poor prognosis. The animal spontaneous tumors are suitable models for human cancer, primarily because both animal population and the tumors are genetically heterogeneous. It provides a new model for future study on prognosis, drug trials and clinical management of breast cancer in women.
